# Structure and properties of the esterase from non-LTR retrotransposons suggest a role for lipids in retrotransposition

**DOI:** 10.1093/nar/gkt786

**Published:** 2013-09-03

**Authors:** Anna M. Schneider, Steffen Schmidt, Stefanie Jonas, Benjamin Vollmer, Elena Khazina, Oliver Weichenrieder

**Affiliations:** ^1^Department of Biochemistry, Max Planck Institute for Developmental Biology, Spemannstrasse 35, 72076 Tübingen, Germany and ^2^Friedrich Miescher Laboratory of the Max Planck Society, Spemannstrasse 39, 72076 Tübingen, Germany

## Abstract

Non-LTR retrotransposons are mobile genetic elements and play a major role in eukaryotic genome evolution and disease. Similar to retroviruses they encode a reverse transcriptase, but their genomic integration mechanism is fundamentally different, and they lack homologs of the retroviral nucleocapsid-forming protein Gag. Instead, their first open reading frames encode distinct multi-domain proteins (ORF1ps) presumed to package the retrotransposon-encoded RNA into ribonucleoprotein particles (RNPs). The mechanistic roles of ORF1ps are poorly understood, particularly of ORF1ps that appear to harbor an enzymatic function in the form of an SGNH-type lipolytic acetylesterase. We determined the crystal structures of the coiled coil and esterase domains of the ORF1p from the *Danio rerio* ZfL2-1 element. We demonstrate a dimerization of the coiled coil and a hydrolytic activity of the esterase. Furthermore, the esterase binds negatively charged phospholipids and liposomes, but not oligo-(A) RNA. Unexpectedly, the esterase can split into two dynamic half-domains, suited to engulf long fatty acid substrates extending from the active site. These properties indicate a role for lipids and membranes in non-LTR retrotransposition. We speculate that Gag-like membrane targeting properties of ORF1ps could play a role in RNP assembly and in membrane-dependent transport or localization processes.

## INTRODUCTION

Non-LTR retrotransposons (retrotransposons like the human LINE-1 element that do not contain long terminal repeats, LTRs) represent a major evolutionary force acting on the structure and composition of eukaryotic genomes (1–5). However, despite their significance for evolution and disease, non-LTR retrotransposons are poorly understood on a mechanistic level, especially if compared with LTR retrotransposons and retroviruses (5–7). Whereas all those retroelements propagate in a ‘copy-and-paste’ fashion via an RNA intermediate, their mechanisms of reverse transcription and genome integration are fundamentally different. Most strikingly, for LTR retrotransposons and retroviruses the reverse transcription takes place in the cytoplasm, in the context of virus-like particles (VLPs) that have a regular scaffold formed by the Gag protein (5–7). Among those, HIV Gag from the human immunodeficiency virus (HIV) is studied best and described as a self-associating multidomain protein that combines both RNA packaging and membrane binding functions (8). In contrast, for non-LTR retrotransposons the reverse transcription takes place in the nucleus and is directly coupled to genomic integration by target-primed reverse transcription (9,10). Furthermore, non-LTR retrotransposons lack homologs of the Gag protein and do not seem to have viral relatives that would allow a horizontal transfer across cell boundaries (5–7).

**Figure 1.**
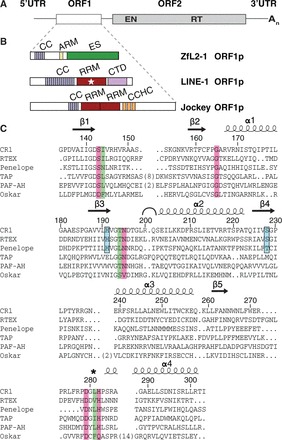
The esterase encoded by non-LTR retrotransposons. (**A**) General organization of an RNA from a non-LTR retrotransposon, encoding a first, accessory ORF1 and a second, catalytic ORF2 that harbors both endonuclease (EN) and reverse transcriptase (RT) functions required for target-primed reverse transcription. (**B**) Class representatives for ORF1ps and their domain architecture. The three structural classes are illustrated by ORF1ps from the *Danio rerio* ZfL2-1 element, the human LINE-1 element and the *Drosophila melanogaster* Jockey element. The unique SGNH-type esterase, ES, is highlighted in green. Coiled coil domains promoting self-association (CC, gray blue) are indicated as well as proposed RNA binding elements: RRM (star) and CTD indicate LINE-1-like RRM and C-terminal domains. RRM and CCHC indicate other RRM domains and Gag-like CCHC zinc knuckles. ARM indicates arginine-rich peptide motifs. (**C**) Structure-based sequence alignment of the ZfL2-1 esterase. Esterases from non-LTR retrotransposons are aligned with crystallized SGNH hydrolases (TAP and PAF-AH, Supplementary Figure S2) and with a non-catalytic SGNH protein (Oskar). Transposon-encoded esterases are from different clades (CR1, RTEX, Penelope) and animal phyla (chordates, cnidarians, sponges). Numbers and secondary structures are from the ZfL2-1 esterase, representing the CR1 clade. An arc indicates the hinge around R200, and an asterisk denotes the remodeled L281 (Figure 3F). Catalytic residues are boxed in magenta, positions of gating residues in green and transposon-specific positions in cyan. See Supplementary Figure S1 for additional details and accession numbers.

Instead of Gag, non-LTR retrotransposons frequently encode a distinct multidomain protein named ORF1p (11–19), which is translated from the first open reading frame (ORF1, Figure 1). A typical ORF1p usually contains one or two RNA recognition motif (RRM) domains (18), and it forms multimers, which is often indicated by the presence of a coiled coil domain (20–23). The RRM domains are thought to mediate RNA binding (18), leading to the formation of RNP retrotransposition intermediates that contain both ORF1p and ORF2p (harboring the reverse transcriptase function), but that lack the regular shape of VLPs (24,25). The RRM domains occur in the context of two distinct classes of ORF1p architecture (18). These are represented in Figure 1B by the LINE-1 ORF1p from the human LINE-1 element (22), and by the Jockey ORF1p from the *Drosophila melanogaster* Jockey element (12). Proteins that belong to the class of LINE-1-like ORF1ps trimerize (21) and can be identified via their distinct RRM domain (18). Crystal structures of such trimers reveal a highly complex architecture, remotely similar to membrane fusion proteins such as HIV-Env/gp41 or the influenza hemagglutinine-esterase (22). Proteins that belong to the class of Jockey-like ORF1ps are characterized by one or two RRM domains immediately followed by one or more CCHC zinc knuckles (18). These are similar to the zinc knuckles in the nucleocapsid domain of HIV Gag (11,12) and thought to cooperate with the RRM domains in the interaction with RNA (18). Intriguingly, some ORF1ps appear to have additional functionality that goes beyond self-association and RNP formation. This is indicated by sequence analyses that suggest the presence of an esterase domain (15), classified as a lipolytic acetylhydrolase of the SGNH family (26,27) (Figure 1).

To learn whether the esterase domain is more than a mere molecular fossil and to learn about the potential functions of esterase-encoding ORF1ps in non-LTR retrotransposition, we took a structure-based approach and characterized the ORF1p of the ZfL2-1 non-LTR retrotransposon from zebrafish, *Danio rerio* (28). In a HeLa cell-based assay (29), the ORF1p was reported to enhance retrotransposition of the ZfL2-1 element, but it is not essential in these cells (28,30). Furthermore, the protein was shown to self-associate and was suggested to interact with RNA (23), although it lacks an apparent RNA binding domain. We defined the boundaries of two functional domains and determined their crystal structures. The first structure is that of an N-terminal coiled coil domain that we show to cause a dimerization of the molecule, an assembly mode that is clearly distinct from the trimers formed by the LINE-1 ORF1p (21,22). The second structure is that of the esterase domain, demonstrating the three-dimensional conservation and an unexpected disposition to accommodate long fatty acid chains. We also demonstrate that the esterase is enzymatically active and binds to negatively charged liposomes. Together, these findings define a third class of ORF1p architecture and suggest that lipids may play a role in non-LTR retrotransposition.

## MATERIALS AND METHODS

### Sample preparation

**Figure 2.**
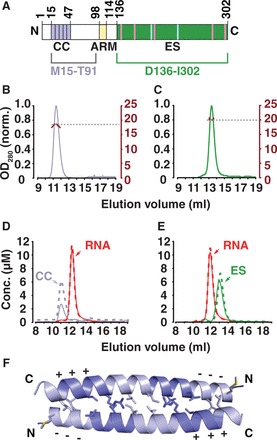
Multimerization and RNA binding properties of the ZfL2-1 ORF1p. (**A**) Domain structure of the ZfL2-1 ORF1p. Domain boundaries (Figure 1B) are numbered, and conserved positions (Figure 1C) are indicated. The soluble and non-aggregating protein fragments used in this work exclude the arginine-rich motif (ARM) and are outlined by brackets. (**B** and **C**) SEC coupled to static MALLS. The N-terminal construct comprising the coiled coil domain (B, residues M15-T91, M_r_ = 9 kDa) elutes as a non-spherical dimer with a hydrodynamic radius (r_H_) of 27 Å and a mass (M_r_) of 18 kDa. The isolated esterase domain (C, residues D136–I302, M_r_ = 19 kDa) elutes as a spherical monomer with a hydrodynamic radius (r_H_) of 11 Å and a mass (M_r_) of 19 kDa. (**D** and **E**) Quantitative SEC. Proteins and RNAs were either injected separately (dotted lines) or as mixtures (solid lines). Neither the N-terminal construct (D), nor the isolated esterase domain (E) forms stable complexes with oligo-(A)_27_ RNA (red). (**F**) Crystal structure of the coiled coil domain (M15-P47). Heptad repeats within the antiparallel dimer are shaded alternatingly and charge compensation between acidic (−−−) and basic (+++) heptads is indicated. Side chains in hydrophobic positions a and d of each heptad repeat are drawn as sticks.

DNA sequences for the expression of the N-terminal construct comprising the coiled coil domain, ZfL2-1_CC (M15-T91, Figure 2A and B), as well as for the esterase domain, ZfL2-1_ES (D136-I302, Figure 2A and C), were amplified by polymerase chain reaction (PCR) from a chemically synthesized and codon-optimized DNA template (Invitrogen), encoding the complete ORF1 protein of the ZfL2-1 element (Uniprot ID Q3LG57) (28). Mutations were generated using QuikChange site-directed mutagenesis PCR (Stratagene). Proteins were expressed from a pETM41P (EMBL) plasmid in *Escherichia coli* BL21-Star cells at 20°C overnight. Purification included maltose binding protein (MBP) and heparin affinity steps followed by size-exclusion chromatography (SEC) into storage buffer (10 mM Tris/HCl, pH = 7.5, 150 mM NaCl).

### Multimerization and RNA binding

Analytical SEC and multi-angle laser light scattering (MALLS) experiments were done in reaction buffer (20 mM Tris/HCl, pH = 7.5, 250 mM NaCl, 5 mM MgCl_2_) on a Superdex 75 (10/300 GL) column that was mounted on an ÄKTA Purifier-10 (GE Healthcare) and followed online by miniDAWN TREOS and Optilab rEX instruments (Wyatt Technologies). The relative contributions of protein and RNA to the total ultraviolet absorption were calculated at each wavelength (simultaneously monitored at 230, 260 and 280 nm) assuming for each substance a constant ratio of its extinction coefficients at 230 and 280 nm (31).

### Activity assay

Hydrolysis of *p*-nitrophenol (pNP) esters was monitored in reaction buffer at 25°C on a Tecan Infinite F200 spectrophotometer by an absorption increase at 405 nm for released pNP (ε = 17 800 l mol^−^^1 ^cm^−^^1^). Initial velocities were plotted as a function of substrate concentration to determine kinetic parameters, K_M_ and k_cat_. For specific activity, 1 U equals a substrate turnover of 1 µmol min^−^^1^. To test for peptide deacetylation we used an histone deacetylase fluorimetric assay kit (Enzo Life Sciences) according to the manufacturer’s instructions.

### Lipid overlay and liposome binding assays

Membrane lipid strips (Echelon Biosciences) contained single lipid species spotted on a hydrophobic membrane (100 pmol per spot) and were incubated with proteins overnight [3× phosphate buffered saline (PBS), 0.3% Tween, 3% bovine serum albumin]. The esterase was used as MBP-His-fusion protein and detected by a specific primary antibody (anti-His, Sigma), followed by a horseradish peroxidase-conjugated secondary antibody.

Liposomes (d = 0.30 µm) were prepared according to the manufacturer’s protocol from polar lipid extract (Avanti Polar Lipids). Protein and liposomes were mixed as described (32), incubated and brought to a sucrose concentration of 32% in a centrifugation tube (2.5–5 µM protein, 150 µl). This was overlaid in two steps by a 14% sucrose cushion and topped by a layer of 1 × PBS. Following a centrifugation step, protein from the top fraction (300 µl) was precipitated and analyzed by sodium dodecyl sulphate polyacrylamide gel electrophoresis.

### Crystallization and data collection

For crystallization in sitting drops (0.4 over 80 µl reservoir), sample in reaction buffer was mixed 1:1 with reservoir. For the coiled coil domain (Figure 2B), a single crystal was found after 4 months over a reservoir of 0.2 M Na-thiocyanate and 20% (w/v) polyethylene glycol (PEG) 3350. The crystal was flash-frozen in liquid nitrogen, from reservoir solution supplemented with 20% glycerol. For the esterase domain (Figure 2C), crystals grew over 0.1 M Na-Hepes, pH = 7.0, 0.5% (v/v) Jeffamine and 1.1 M Na-malonate. A heavy atom derivative was obtained by an overnight incubation in reservoir supplemented with 2 mM potassium dicyanoaurate. Crystals were flash-frozen from reservoir solution. Diffraction data were collected on beamline PXII of the Swiss Light Source, Villigen, Switzerland, and diffraction images were processed using XDS (33).

### Crystal structure solution, refinement and modeling

The structure of the coiled coil domain was solved by molecular replacement using MOLREP (34) from within the CCP4 package (35) and a polyalanine model of an anti-parallel coiled coil as a search model, derived from PDB-ID 1A92 (36). The structure of the esterase domain was solved by single isomorphous replacement with anomalous scattering (SIRAS). SHELX C/D/E was used to identify the sites for phasing and for an initial auto-building of the structure (37,38). Both structures were then (re-)built automatically also to remove any potential model bias in the case of the coiled coil domain, using ARP/wARP (39) and BUCCANEER (40). The model was finished manually in COOT (41), alternating with rounds of refinement using PHENIX (42). Final refinement rounds were done in PHENIX, refining TLS parameters in addition to individual B-factors and including hydrogens. Stereochemical properties were analyzed with MOLPROBITY (43). Modeling of the rotated L281 and of the enclosed palmitate in the context of the closed esterase monomer (ZfES_BA, connecting residues M135-L199 from chain B with residues R200-I302 from chain A) was also achieved in COOT followed by energy minimization in PHENIX. Cavity volumes were extracted using the Voss Volume Voxelator (44) using inner and outer probe radii of 1.2 and 5.0 Å, respectively. Figures were generated in PyMOL (http://pymol.org/) using the APBS plug-in (45) to visualize electrostatic surface potentials.

## RESULTS

### Esterases are found in distinct clades of non-LTR retrotransposons and share specific properties

Non-LTR retrotansposons (Figure 1A) are thought to be acquired primarily by vertical transmission and were originally grouped into clades of distinct domain composition that date back to the Precambrian era (7). For the classification of newly identified non-LTR retrotransposons, consensus sequences are deposited in RepBase (46) and placed into their respective clade according to sequence alignments of the reverse transcriptase (17).

We retrieved ORF1p sequences containing an SGNH esterase from RepBase and aligned them with SGNH proteins of known structure (Figure 1C and Supplementary Figure S1). The alignment reveals that the esterase is not limited to the CR1 clade, where it was originally identified (15), but can also be found in members of the RTE clade, RTEX (17) and even in Penelope-like elements, the most deeply branched clade of non-LTR retrotransposons (19). Also, esterase-containing non-LTR retrotransposons are not limited to certain species, but are distributed over many animal phyla.

Most importantly, despite the low sequence identity, the residues constituting the active site have been strongly conserved. This suggests a beneficial role of esterase structure and enzyme function for the propagation of the respective non-LTR retrotransposons. Moreover, the alignment reveals additional positions (H191, S228) that are conserved only among the transposon-encoded esterases, pointing at specialized transposon-specific properties and a monophyletic origin (Figure 1C and Supplementary Figure S1), despite the scattered presence of the esterase in different clades and species.

### Esterase-containing ORF1ps can form multimers but lack structural RNA binding domains

Further analysis of the esterase-containing ORF1ps reveals that the esterase domain is generally preceded by sequences predicted to form coiled coils and connected to these sequences by poorly conserved linkers (Supplementary Table S1). The presence of coiled coil domains indicates these proteins to form multimeric assemblies, as observed for many previously characterized ORF1ps (20–23). Interestingly, none of the esterase-containing ORF1ps in RepBase included RRM domains or any other structural domain known to bind RNA. It hence appears that such a domain combination never occurred in the past or that it bears no selective advantage, for example, because it is functionally redundant or even mutually exclusive. In either case, the observation raises the question how the esterase-containing ORF1ps could interact with RNA and become part of stable RNPs.

We therefore decided to express and purify the ZfL2-1 ORF1p (28) and to characterize its properties *in vitro* (Figure 2). We obtained soluble material for an N-terminal construct comprising the coiled coil domain and for the C-terminal esterase domain (Figure 2A–C). In analytical SEC, the N-terminal construct indeed shows self-interaction (23) and we find it to exclusively form dimers, whereas the esterase domain remains monomeric at concentrations up to 75 µM (Figure 2B and C). Furthermore, the two constructs did not interact with each other and failed to form stable and separable complexes with single-stranded oligo-(A)_27_ RNA (Figure 2D and E) under conditions previously used for the LINE-1 ORF1p (22).

Consequently, the separated domains and the highly positive charge of the esterase alone (pI = 10.8) are not sufficient for a general activity to bind single-stranded RNA. However, we needed to exclude the arginine-rich motif (ARM, Figure 1B) between amino acids G98 and T114 of the ZfL2-1 ORF1p to obtain soluble and non-aggregating protein constructs. Such motifs are frequently found in members of the CR1 clade (Figure 2A and Supplementary Table S1), and it has been described that an N-terminal fragment of the ZfL2-1 ORF1p that includes the ARM can be photocrosslinked to RNA *in vitro* and that it helps to remodel nucleic acid structures (23). We therefore speculate that the esterase-containing ORF1ps could bind their RNA messengers not via structural protein domains, but rather via positively charged peptides that target specific structural RNA elements, as described for the λ-N peptide (47).

### The coiled coil domain of the ZfL2-1 ORF1p crystallizes as an antiparallel dimer

A proteolytically stable fragment of the N-terminal construct gave crystals that contained two copies of an antiparallel homodimeric coiled coil per asymmetric unit, and the structure was refined to an R_free_ of 21.5% at 1.55 Å resolution (Supplementary Table S2). Each α-helix comprises five heptad repeats, confining the coiled coil to the region between M15 and P47 as we would predict from the sequence. Because of the charge compensation between the highly acidic N-terminal heptads and the highly basic C-terminal heptads, we presume a preference for the antiparallel orientation also in the absence of crystal packing constraints (Figure 2F), an arrangement that is clearly distinct from the parallel trimeric coiled coil formed by the LINE-1 ORF1p (22). Whereas coiled coil domains and their ability to multimerize are hence frequently present among ORF1ps, the resulting assemblies can differ significantly in terms of structure and likely do not always have a common evolutionary origin.

### The esterase domain of the ZfL2-1 ORF1p crystallizes as two dynamic half-domains

**Figure 3.**
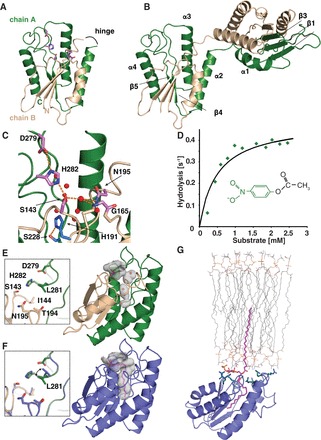
Crystal structure and activity of the ZfL2-1 esterase. (**A** and **B**) Structural overview. The esterase consists of two half-domains (green and wheat) connected by a flexible hinge (A) that allows it to crystallize as a domain-swapped dimer (B). Secondary structure elements are indicated, and conserved catalytic residues are shown as sticks (magenta). (**C**) Details of the active site. Transposon-specific residues are included as sticks (cyan), waters are shown as red spheres and hydrogen bonds as dotted lines. (**D**) Enzymatic activity. Representative Michaelis–Menton curve using pNP actetate (inset) as a substrate. K_M_ = 0.43 ± 0.10 mM, k_cat_ = 0.32 ± 0.07 s^−1^, k_cat_/K_M_ = 740 ± 240 s^−1^M^−1^; standard error from six independent experiments. (**E**) Energy-minimized monomer (gate closed). The active site is marked by a superimposed acetate (magenta sticks, from Supplementary Figure S2D). Internal, water-filled cavities and the active site are shown as inverted surfaces. They are separated by the gating residues I144, T194 and L281 (inset). (**F**) Energy-minimized monomer (gate opened) in presence of a palmitate. The deep invagination accommodating the palmitate (magenta sticks) results from a simple rotation of L281 that opens the gate. Placement of the palmitate was guided by the enclosed waters and superimposed octanoic acid (from Supplementary Figure S2B). The inset shows an overlay of the closed and opened gate. (**G**) Model for Gag-like membrane binding via a sequestered phospholipid [adapted from (52)]. Arginines and lysines are shown as blue sticks and thought to cause an electrostatic attraction. The sequestered palmitate is as in (F).

The structure of the ZfL2-1 esterase (Figure 3) was solved by SIRAS and refined at 2.5 Å resolution to an R_free_ of 21.0% (Supplementary Table S3). As expected for an SGNH hydrolase, the protein adopts a flavodoxin-like fold with a central sheet of five parallel β-strands sandwiched by α-helices on both sides (Figure 3A and B). The ZfL2-1 esterase is highly similar to well-characterized SGNH hydrolases (Supplementary Figure S2). These include thioesterase I/protease I/lysophospholipase L1 (TAP) (48) from *Escherichia coli* that can remove 1-acyl groups from lysophospholipids, and cytosolic platelate activating factor acetylhydrolase (PAF-AH) (49) from bovine brain that can remove the 2-acetyl group from PAF and has been implicated in lissencephaly (50). Compared with TAP and PAF-AH, the identity and position of the catalytic residues are highly conserved in ZfL2-1. These residues include D279, H282 and S143, which form the catalytic triad, as well as N195 and G165 that line the oxyanion hole (Figure 3C and Supplementary Figure S2A–D).

The crystal packing of the ZfL2-1 esterase is of particular interest because the protein crystallized as a domain-swapped dimer with three molecules (i.e. 1.5 dimers) per asymmetric unit (Figure 3B). As a consequence, each esterase domain in the crystal is composed of two fragments; an N-terminal half (residues 135–199) with ββαβ topology that is complemented by a C-terminal half (residues 200–302) with αβαβα topology from the neighboring molecule. Because the linker between the two halves is short and close to the 2-fold axis of the dimer, a minimal rearrangement of residues R200 and Q201 is sufficient to reconnect the two chains and to obtain a model for the monomer (hinge, Figure 3A).

Although in solution we only detect the monomeric form, the observed crystal packing demonstrates that the esterase can apparently split open into two halves at least transiently, disengaging β-strands β3 and β4 of the central β-sheet and tearing apart the network of hydrogen bonds in the active site (Figure 3C). These dynamics are facilitated by the fact that the core of the esterase is not entirely hydrophobic and even contains enclosed water-filled cavities that require structural rearrangements to gain access to the solvent (Figure 3E and F). Most interestingly, the hydrogen bonds between the two half-domains also include an interaction between the transposon-specific residues H191 and S228 deep inside the core of the esterase (Figure 3C). This indicates that the composition of two reversibly separable half-domains is likely conserved among transposon-encoded esterases and has functional significance.

### The ZfL2-1 esterase shows hydrolytic activity

In the crystal of the ZfL2-1 esterase, residues I144, T194 and L281 confine the size of the active site and separate it from the internal cavities (Figure 3E). Based on the comparison with the structure of PAF-AH, these residues are thought to limit the fatty acid moiety of the ester substrate to short carbon chains, whereas the alcoholic moiety of the ester substrate faces the solvent and probably contributes only marginally to substrate specificity (Supplementary Figure S2B and D). Therefore, we used pNP acetate as an ester substrate analog and monitored hydrolysis by absorption change. As expected from the structure, we find the ZfL2-1 esterase to be enzymatically active (Figure 3D). The activity crucially depends on the active site of the enzyme because individual point mutations of the catalytic residues (S143A/Y/Q, H282S) completely abolish hydrolysis. In addition, activity is blocked by a S228A/H191F double mutation of the two transposon-specific residues (Figure 1C and 3C), indicating the internal network of hydrogen bonds to be relevant also for the catalysis. However, the catalytic turnover, k_cat_, and hence the specific activity of the ZfL2-1 esterase domain is significantly lower than reported for most other SGNH hydrolases (0.92 U mg^−^^1^, as compared with 345 U mg^−^^1^ for TAP) (27), suggesting that it is not optimized for catalytic turnover. Furthermore, because many SGNH hydrolases have a rather broad range of substrate specificity (27), we also tested for phosphoesterase and peptidase activities. However, we failed to detect any activity, neither with pNP phosphate as a substrate, nor using a standard kit for the deacetylation of histone-derived peptides. Finally, regarding the length of the fatty acid chain, pNP esters such as pNP butyrate are hydrolyzed as well (estimated catalytic efficiency, k_cat_/K_M_ = 590 s^−^^1 ^M^−^^1^), but a quantitative analysis of pNP esters with even longer fatty acid chains is precluded by their limited solubility.

### Long fatty acid chains could reach and fill the space between the half-domains

A comparison of the three independent protein copies in the crystal reveals that the side chains of I144 and L281 are flexible and can adopt alternative conformations, suggesting that they could act as gating residues between the active site and the internal cavities (Supplementary Figure S2E and F). Indeed, a simple rotation of L281 would cause the gate to open up and connect the internal cavities to the surrounding solvent. As a consequence, the volume accessible to the fatty acid would increase from ∼50 to 270 Å^3^ and hence would allow the accommodation of longer fatty acid chains such as the ones found in membrane phospholipids (Figure 3E and F).

However, it is difficult to imagine how the carbon chain would thread through the gate and displace the internal water molecules. We therefore suggest that it enters laterally and gets engulfed between the two half-domains when they move apart, turning around the described hinge at R200–Q201 as proposed above for the formation of the domain-swapped dimer (Figure 3A and B). This binding mechanism should be clearly facilitated in the context of the full-length protein, where two esterase domains are closely tethered together by the coiled coil. It would probably help to access phospholipid substrates at the surface of cellular membranes by ‘interfacial activation’ (51), resulting in membrane binding topologies that are comparable with the one proposed for the interaction of the matrix (MA) domain of HIV Gag with phosphatidylinositol 4,5-bisphosphate (52). Similar to the MA-domain, the putative membrane-facing surface of the ZfL2-1 esterase is rather flat and displays a striking accumulation of positively charged arginine and lysine residues (Figure 3G).

### The ZfL2-1 esterase binds negatively charged phospholipids and liposomes

**Figure 4.**
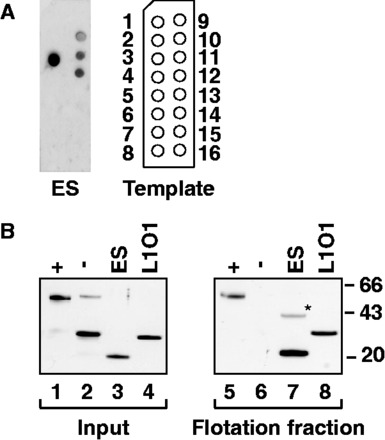
Interaction of the ZfL2-1 esterase with phospholipids and liposomes. (**A**) Lipid overlay assay. An MBP-His-tagged version of the ZfL2-1 esterase (ES) was incubated with membrane lipid strips under stringent salt conditions (3× PBS), and bound protein was detected by immunostaining. The positions of the spotted lipids are as follows: 1, triglyceride; 2, diacylglycerol; 3, phosphatidic acid; 4, phosphatidylserine; 5, phosphatidylethanolamine; 6, phosphatidylcholine; 7, phosphatidylglycerol; 8, cardiolipin; 9, phosphatidylinositol (PI); 10, PI-[4]-phosphate (PI[4]P); 11, PI[4,5]P_2_; 12, PI[3,4,5]P_3_; 13, cholesterol; 14, sphingomyelin; 15, [3]-sulfogalactosylceramide; 16, blank. The esterase preferentially interacts with phosphatidic acid and the three phosphatidylinositol phosphates. MBP-His alone or GST did not show any signal. (**B**) Flotation experiment using liposomes. Silver-stained gels show input samples (20%, left panel) and samples recovered from the floating liposomes (right panel). Lanes 1, 5: (+), positive control, Nup133 [residues 67–514, (32)], a nucleoporin. Lanes 2, 6: (−) negative control, GST. Lanes 3, 7: ES, ZfL2-1 esterase (residues 136–302). Lanes 4, 8: L1O1, LINE-1 ORF1p [residues 104–337, (22)]. The asterisk denotes a weak ES dimer in the flotation fraction, a likely gel separation artifact.

To test whether the ZfL2-1 esterase is able to gain access to phospholipids at membrane surfaces independently of other proteins, we first did an assay with commercially available membrane lipid strips, where proteins that bind prespotted lipids on a solid support are detected by antibody staining (Figure 4). Under stringent salt conditions the ZfL2-1 esterase exclusively binds a selection of negatively charged phospholipids, including phosphatidic acid and phosphatidyl-inositol phosphates (Figure 4A).

This assay was followed by an established liposome flotation assay with liposomes prepared from polar lipid extracts [Supplementary Figure S3, (32)]. Here, we find the ZfL2-1 esterase to migrate through a sucrose gradient together with the liposomes, similarly to a truncated nucleoporin, Nup133, that served as a positive control, and in contrast to glutathione-S-transferase (GST), which served as a negative control (Figure 4B). Importantly, liposome binding depends on the integrity of the protein structure because heat-denatured ZfL2-1 esterase no longer co-purifies with the floating liposomes. However, neither our active site point mutations nor the H191F/S228A double mutation prevented liposome binding (Supplementary Figure S3B and C), suggesting that initial membrane surface binding as tested in the liposome flotation assay is primarily driven by protein surface charge (Supplementary Figure S3D) and does not require the sequestration of a fatty acid chain.

Together, these experiments suggest that the esterase domain can indeed target the ZfL2-1 ORF1p to negatively charged membranes in the cell, suggesting a role for membranes in the propagation of non-LTR retrotransposons. Intriguingly, this role may even extend beyond the esterase-encoding elements because a truncated version of the trimeric human LINE-1 ORF1p (22) that starts in the middle of the coiled coil domain (Figure 1B) also co-purifies with liposomes in our assay (Figure 4B).

## DISCUSSION

The detailed analysis of the ZfL2-1 ORF1p and the comparison with its relatives from other non-LTR retrotransposons establish the esterase-containing ORF1ps as a separate architectural class that lacks defined RNA binding domains (Figure 1). Nevertheless, ZfL2-1-like ORF1ps show functional analogy with LINE-1-like and Jockey-like ORF1ps in RNP assembly because of their self-association via coiled coil domains and because of their ability to facilitate structural rearrangements of nucleic acids *in vitro* (20,23,53). Additional support for a functional analogy among structurally diverse ORF1ps comes from the fact that retrotransposons from within a given clade [as defined by closely related reverse transcriptases (17)] can harbor ORF1ps from different architectural classes (18). This observation not only hints at similar functions of the diverse ORF1ps but also at an apparent ‘exchange’ of ORF1ps between otherwise closely related non-LTR retrotransposons. Such an ‘exchange’ could take place by recombination or mutual cross-insertion of retrotransposons within a common host, or, alternatively, by a consecutive loss and gain of domains. The latter possibility is facilitated by the fact that the loss of transposon-encoded protein domains does not necessarily lead to the extinction of the respective non-LTR retrotransposon because the lost function might still be available ‘in trans’, i.e. from another retrotransposon or from the host. Indeed, this idea has been promoted previously for the apparent loss and gain of the RNaseH domain in non-LTR retrotransposons and retroviruses (54,55), as it can also reconcile the apparently monophyletic origin of protein domains such as the esterase and RNaseH with the scattered distribution across clades and species.

The precise role of the ZfL2-1 ORF1p in retrotransposition is still difficult to address. Although the ZfL2-1 element has been shown to retrotranspose in human HeLa cells (28), its ORF1p is dispensable in this assay (30), and therefore HeLa cells are not expected to reveal ZfL2-1 ORF1p-specific functions. In the context of the natural zebrafish host, however, where the ZfL2-1 element needs to retrotranspose in germ line cells to be passed on to the next generation, the ORF1p may be an important factor. Indeed, the presence of the esterase and the conservation of its transposon-specific properties strongly suggest additional functions for ORF1ps in non-LTR retrotransposition that go beyond their known properties in RNP assembly. The crystal structure of the ZfL2-1 esterase and the positive activity assay demonstrate that the respective domains in related ORF1ps are *bona fide* SGNH hydrolases, and are therefore unique among ORF1ps in harboring an enzymatic function. SGNH hydrolases represent a large enzyme family comprising several thousand members from all domains of life, including viruses. However, even for well-characterized members, such as PAF-AH, the physiological substrates and functions largely remain obscure (26,27). The primary targets are presumably carboxyesters, but some family members also hydrolyze thioesters and even isopeptide bonds (27,48). Most intriguingly, the active sites of certain SGNH proteins have dual binding and enzymatic functions (56) or have entirely lost their hydrolytic activity, such as the homologs of PAF-AH in insects (57) or the Oskar protein (58) in *Drosophila* germ cell formation (Figure 1C and Supplementary Figure S1). One can therefore imagine several mechanisms how an SGNH protein could affect retrotransposition. These include the deacetylation of carbohydrate substrates, lipid messengers or regulatory proteins (50,56,59). Another attractive possibility that requires access to the internal cavities is the reversal of protein palmitoylation controlling membrane association of the target (60). However, there are no known protein targets so far, and we failed to detect activity in a histone deacetylation assay.

Considering the common properties of ORF1ps in the formation of RNPs, and based on our lipid binding assays, we therefore favor a role of the esterase domain in membrane targeting, combined with a potentially regulated phospholipase activity. In this scenario, initial targeting of membrane surfaces would be initiated by a charge-mediated interaction, and would be consolidated by the sequestration of an acyl chain in the space between the two dynamic half-domains, resulting in a membrane binding topology similar to the one proposed for the MA-domain of HIV Gag [Figure 3G, (52)]. Subsequent steps of the retrotransposition cycle could then be regulated by a controlled substrate hydrolysis, releasing both surface-assembled RNPs and lysophospholipid, i.e. phospholipids that lack one of their long fatty acid chains. This release only requires a single round of substrate hydrolysis, consistent with the observation that the ZfL2-1 esterase is apparently not optimized for rapid enzymatic turnover. Lysophospholipids can influence the spontaneous curvature and bending elasticity of phospholipid membranes and have been implicated in the formation of membrane vesicles and other specialized membrane structures (61–63). As an additional consequence of the described process, the esterase-containing ORF1ps could therefore not only drive RNP assembly on membrane surfaces, but could also help to include RNPs in membrane vesicles capable of transferring them across membranes (64,65). On a speculative note, such vesicles might even be implicated in the rare instances where non-LTR retrotransposons like the BovB element transfer horizontally between species (66,67). BovB is a member of the RTE clade that, in contrast to RTEX, encodes only rudimentary ORF1ps or lacks them altogether (68).

The suggested mode for the esterase to associate with membranes is clearly inspired by the HIV Gag protein, which uses membranes to self-assemble into retroviral capsids (8). Furthermore, there is a growing body of data from positive-strand RNA viruses that use virally encoded proteins to assemble RNA-replication complexes at specifically induced membrane structures (69). These parallels between retroviruses and positive-strand RNA viruses raise the question how non-LTR retrotranspons fit into the picture and whether the functional analogy that is emerging between ORF1ps could be extended to membrane-related functions. Therefore, we believe that it is worth further exploring the role of membranes in non-LTR retrotransposition, not only in the context of ZfL2-1-like ORF1ps, but also in the context of the trimeric LINE-1-like ORF1ps with their structural similarity to membrane fusion proteins (22), and in the context of the Jockey-like ORF1ps with their Gag-like CCHC knuckles. The respective studies shall provide important mutual insight into the mechanisms of non-LTR retrotransposition, in particular into hitherto unexplored processes such as the regulation and localization of RNP assembly or into the roles of membranes in RNP transport.

## ACCESSION NUMBERS

The atomic coordinates and structure factors for the ZfL2-1 ORF1p coiled coil domain and for the ZfL2-1 ORF1p esterase were deposited at the protein data bank (PDB) under ID codes 4c1a and 4c1b, respectively.

## SUPPLEMENTARY DATA

Supplementary Data are available at NAR Online including [70,71].

## FUNDING

The Max Planck Society; and Supported by a PhD fellowship from the Boehringer Ingelheim Fonds (to A.M.S.). Funding for open access charge: The Max Planck Society.

*Conflict of interest statement*. None declared.

## Supplementary Material

Supplementary Data
