# Supplementary material for: Structure and properties of the esterase from non-LTR retrotransposons suggest a role for lipids in retrotransposition
Source: Nucleic Acids Res. 2013 Sep 3;41(22):10563–72. doi: 10.1093/nar/gkt786 (PMC3905857; doi:10.1093/nar/gkt786)
Supplement: Supplementary Data [file supp_41_22_10563__index.html]

Structure and properties of the esterase from non-LTR retrotransposons suggest a role for lipids in retrotransposition — Supplementary Data 

# Structure and properties of the esterase from non-LTR retrotransposons suggest a role for lipids in retrotransposition

## Supplementary Data

files

**Files in this Data Supplement:**

- Supplementary Data - pdf file
