# Supplementary material for: Structure and properties of the esterase from non-LTR retrotransposons suggest a role for lipids in retrotransposition
Source: Nucleic Acids Res. 2013 Sep 3;41(22):10563–72. doi: 10.1093/nar/gkt786 (PMC3905857; doi:10.1093/nar/gkt786)
Supplement: Supplementary Data [file supp_gkt786_Schneider_AM_SI_r2.pdf]

Structure and properties of the esterase from non-LTR retrotransposons suggest a role for lipids in retrotransposition

Anna M. Schneider[1], Steffen Schmidt[1], Stefanie Jonas[1], Benjamin Vollmer[2], Elena Khazina[1] and Oliver Weichenrieder[1 *]

[1] Department of Biochemistry, Max Planck Institute for Developmental Biology, Spemannstrasse 35, 72076 Tübingen, Germany

[2] Friedrich Miescher Laboratory of the Max Planck Society, Spemannstrasse 39, 72076 Tübingen, Germany

* To whom correspondence should be addressed. Tel: +4970716011358; Fax: +4970716011353; Email: oliver.weichenrieder@tuebingen.mpg.de

- SUPPLEMENTARY INFORMATION -

Supplementary Tables S1-S3

Supplementary Figures S1-S3

References

Table S1

Domain boundaries

Name	CC-start [a, b]	CC-end [a, b]	ARM-start [a, c]	ARM-end [a, c]	ES-start [a, d]	ES-end [a, d]
CR1-1_DR	M027	K082	G124	T140	D162	T327
CR1-16_DR	n.a.	n.a.	G047	S063	K109	N273
CR1-3_DR	E063	P097	E133	R149	R223	H379
CR1_1a_XT	L073	M120	V132	E148	Q203	E368
CR1-2_XT	T035	L076	D095	G111	Q166	E331
CR1_AC_1	n.a.	n.a.	n.a.	n.a.	Q068	R232
CR1-X1_Pass	F063	R111	E144	P160	R217	R381
CR1-J2_Pass	T077	S118	G150	V166	R220	R384
CR1-L1_Tgu	S043	L091	G116	S132	R187	R351
CR1-K1_Tgu	P104	L152	G181	S132	R252	R461
RTEX-3_NV	N148	K261	n.a.	n.a.	P371	L538
RTEX-2_NV	N151	T264	n.a.	n.a.	P354	L524
RTEX-1_NV	Y151	E261	n.a.	n.a.	N369	G540
RTEX-14_BF	V266	G429	n.a.	n.a.	E496	A664
RTEX-3_BF	Q292	T448	n.a.	n.a.	E529	P697
RTEX-11_BF	D306	K436	n.a.	n.a.	E505	I673
RTEX-5_BF	T276	S382	n.a.	n.a.	Q469	S638
RTEX-2_BF	D356	S411	n.a.	n.a.	D525	S692
RTEX-1_BF	n.a.	n.a.	n.a.	n.a.	V001	P163
RTEX-10_BF	E331	V462	n.a.	n.a.	R550	K722
RTEX-4_BF	N332	C439	n.a.	n.a.	R526	D697
RTEX-1_SK	I284	A447	n.a.	n.a.	Q481	P640
Neptune1_Ren	n.d.	n.d.	n.a.	n.a.	S383	S538
BRIDGE1_FR	n.d.	n.d.	n.a.	n.a.	K242	T399

n.a. = not applicable, n.d. = not detectable

a, amino acid numbers are counted from the last preceding stop codon

b, positions of coiled coil domains (CC) determined according to COILS (70)

c, positions of arginine-rich motifs (ARM) determined by local sequence alignment of elements from the CR1 clade alone

d, positions of esterase domains (ES) determined from the sequence alignment in Supplementary Figure S1

Table S2

Data collection and refinement statistics for the ZfL2-1 ORF1p coiled coil domain

**Dataset**	**native**	
**Data collection**		
Wavelength, Å	0.97138	
Resolution Range, Å	37 - 1.55	
Space Group	$P2_1$	
Unit Cell		
dimensions (a / b / c), Å	23.7 / 52.4 / 51.8	
angles (α / β / γ), °	90 / 99.8 / 90	
R_{merge}, %	5.5	(47.4)*
Completeness, %	95.6	(93.3)*
Multiplicity	2.5	(2.5)*
I / σ(I)	10.3	(2.0)*
**Refinement**		
R_{work}, %	18.6	
R_{free}, %	21.5	
Number of reflections	17443	
Number of molecules per asymmetric unit		
protein molecules	4	
atoms (excluding water)	1143	
water molecules	116	
ligand atoms	30	
Average B-factor (isotropic), $Å^2$	25.8	
Ramachandran plot		
most favored regions, %	100	
disallowed regions, %	0	
R.m.s.d. from ideal geometry		
bond lengths, Å	0.013	
bond angles, °	1.39	

* Values in parentheses correspond to those in the outer resolution shell (1.59 Å - 1.55 Å)

Table S3

Data collection and refinement statistics for the ZfL2-1 ORF1p esterase

**Dataset**	**native**		**$KAu(CN)_2$**	
**Data collection**				
Wavelength, Å	1.0000		1.0397	
Resolution Range, Å	46 - 2.50		47 - 2.58	
Space Group	$I4_1$		$I4_1$	
Unit Cell				
dimensions (a / b / c), Å	111.3 / 111.3 / 115.7		110.9 / 110.9 / 116.8	
angles (α / β / γ), °	90 / 90 / 90		90 / 90 / 90	
R_{merge}, %	6.6	(66.3)*	8.6	(57.2)*
Completeness, %	100	(100)*	99.4	(93.6)*
Completeness (anomalous), %			96.6	(89.9)*
Multiplicity	6.9	(7.1)*	6.4	(6.4)*
Multiplicity (anomalous)			3.4	(3.4)*
I / σ(I)	19.1	(3.0)*	13.3	(2.6)*
**Refinement**				
R_{work}, %	16.7			
R_{free}, %	21.0			
Number of reflections	24346			
Number of molecules per asymmetric unit				
protein molecules	3			
atoms (excluding water)	4025			
water molecules	74			
Average B-factor (isotropic), $Å^2$	51.6			
Ramachandran plot				
most favored regions, %	96.1			
disallowed regions, %	0.0			
R.m.s.d. from ideal geometry				
bond lengths, Å	0.011			
bond angles, °	1.18			

* Values in parentheses correspond to those in the outer resolution shell (2.56 Å - 2.50 Å)

Figure S1

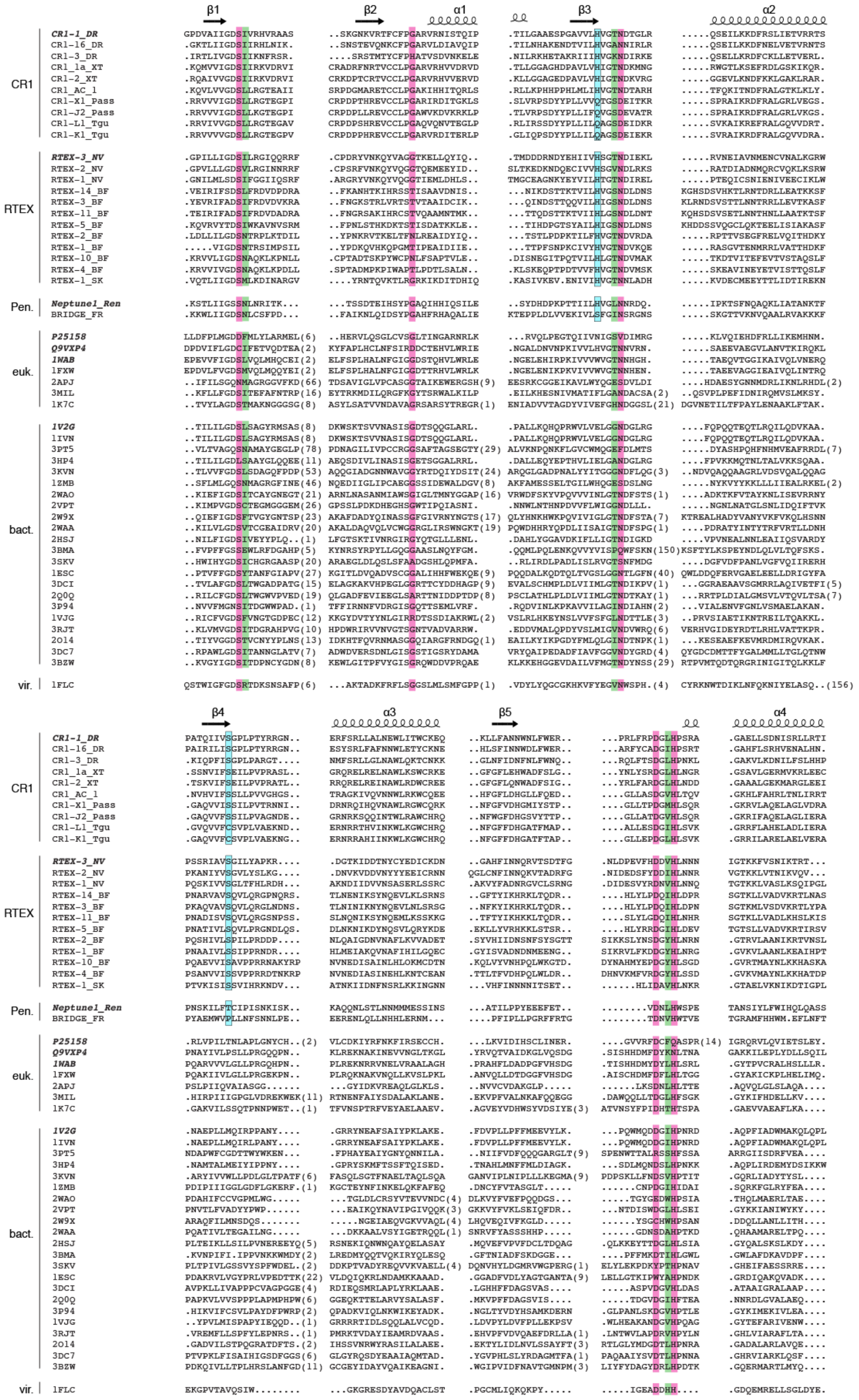

Figure S1. Extended structure-based sequence alignment of esterases from non-LTR retrotransposons with other SGNH proteins.

Esterases from non-LTR retrotransposons are listed by their RepBase ID (46), crystallized SGNH proteins by their PDB ID, and other, non-catalytic SGNH proteins by their Uniprot ID. Non-LTR retrotransposon clades are indicated: CR1, RTEX, and Penelope (Pen.). Animal phyla include chordates (BF, *Branchiostoma floridae*; DR, *Danio rerio*; FR, *Takifugu rubripes*; XT, *Xenopus tropicales*; AC, *Anolis carolinensis*; Pass, *Passeriformes*; Tgu, *Estrildidae*), hemichordates (SK, *Saccoglossus kowalevskii*), cnidarians (NV, *Nematostella vectensis*), and sponges (Ren, *Amphimedon queenslandica*). The other SGNH proteins are grouped according to their origin as eukaryotic (euk.), bacterial (bact.), and viral (vir.). Identifiers of sequences used in Figure 1C are highlighted in bold italics: CR1, CR1-1_DR (ZfL2-1); RTEX, RTEX-3_NV; Penelope, Neptune1_Ren; TAP, 1V2G (*Escherichia coli*); PAF-AH, 1WAB (*Bos taurus*); Oskar, P25158 (*Drosophila melanogaster*). Additional, non-catalytic SGNH proteins are PAF-AH homologs from insects (57), represented by the sequence from *Drosophila melanogaster* (Q9VXP4). The secondary structure assignment corresponds to the crystal structure of the ZfL2-1 esterase. Longer insertions are deleted, including the large insertion between the two half-domains of the viral esterases (number of residues in brackets). Catalytic residues are boxed in magenta, positions of gating residues in green, and transposon-specific positions in cyan.

Figure S2

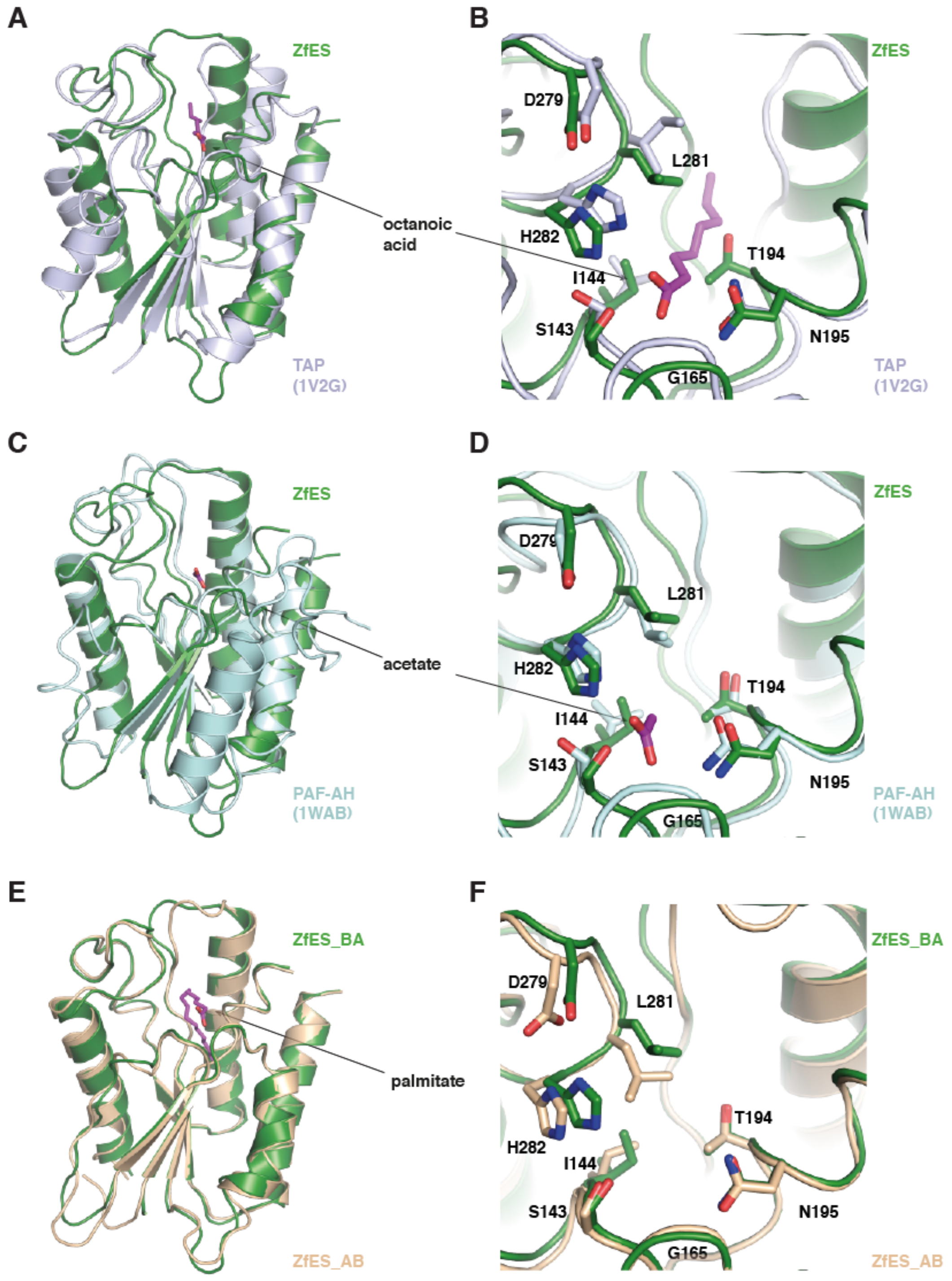
A
ZfES
octanoic
acid
TAP
(1V2G)
B
ZfES
D279
L281
H282
T194
I144
S143
N195
G165
TAP
(1V2G)
C
ZfES
acetate
PAF-AH
(1WAB)
D
ZfES
D279
L281
H282
T194
I144
S143
N195
G165
PAF-AH
(1WAB)
E
ZfES_BA
palmitate
ZfES_AB
F
ZfES_BA
D279
L281
H282
T194
I144
S143
N195
G165
ZfES_AB

Figure S2. Structural comparison of the ZfL2-1 esterase

(**A, B**) Superposition of the ZfL2-1 esterase (ZfES, green) with the TAP protein from *Escherichia coli* (PDB ID: 1V2G) (48). The overview (A) results from a structure-based DALI search that produced a Z-score of 16.8 and an r.m.s.d. value of 2.6 Å over 178 C_α atoms. The details of the active site (B) reveal the structural conservation of the catalytic residues and the accommodation of an octanoic acid (magenta) in TAP together with the orientation of the gating residues.

(**C, D**) Superposition of the ZfL2-1 esterase (ZfES, green) with the PAF-AH protein from bovine brain cells (PDB ID: 1WAB) (49) The overview (C) results from a structure-based DALI search that produced a Z-score of 19.6 and an r.m.s.d. value of 2.0 Å over 212 C_α atoms. The details of the active site (D) reveal the structural conservation of the catalytic residues and the accommodation of an acetic acid (magenta) in PAF-AH together with the orientation of the gating residues.

(**E, F**) Superposition of two crystallographically independent monomers of the ZfL2-1 esterase (ZfES_BA from Figure 3A, green, and ZfES_AB from the same domain-swapped dimer, Figure 3B). The overview (E) reveals regions of conformational plasticity and corresponds to an r.m.s.d. value of 1.22 Å over 167 C_α atoms. The modeled palmitate (Figure 3F, magenta) is included for orientation. The details of the active site (F) illustrate its flexibility, in particular of the gating residues I144 and L281.

Residues are shown as sticks with oxygens in red and nitrogens in blue.

Figure S3

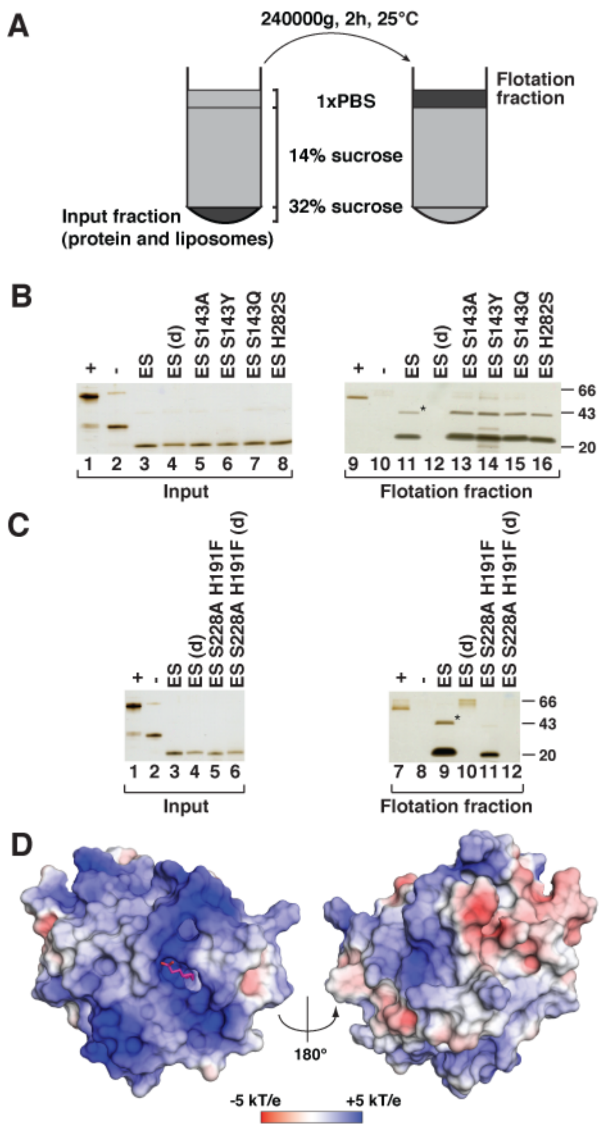
A
240000g, 2h, 25°C
Flotation fraction
1xPBS
14% sucrose
32% sucrose
Input fraction (protein and liposomes)
B
+
-
ES
ES (d)
ES S143A
ES S143Y
ES S143Q
ES H282S
66
43
20
1 2 3 4 5 6 7 8
Input
9 10 11 12 13 14 15 16
Flotation fraction
C
ES S228A H191F
ES S228A H191F (d)
1 2 3 4 5 6
7 8 9 10 11 12
D
180°
-5 kT/e
+5 kT/e

Figure S3. Liposome flotation assay

(**A**) Scheme of the flotation assay (32,71). Protein and liposomes are gently mixed and agitated (30 min, 25°C, 600 rpm), and brought to a sucrose concentration of 32 % in a centrifugation tube (d = 11 mm, 1 x PBS, 2.5 - 5 µM protein, 150 µl). This input fraction is overlaid in two steps by a 14 % sucrose cushion (2 x 850 µl) and topped by a layer of 1 x PBS (300 µl). Following a centrifugation step (2 h, 25° C, 240000 g), protein from the top, flotation fraction (300 µl) contains liposomes that rose through the sucrose gradient, together with potentially attached lipid binding proteins.

(**B, C**) Characterization of the liposome binding properties of the ZfL2-1 esterase. Silver-stained gels show input samples (left panel, 20 %) and samples recovered from the floating liposomes (right panel). Positive (+) controls (Nup133, residues 67-514) and negative (-) controls (GST) are as in Figure 4B. Single point mutations of catalytic residues do not affect liposome binding. Compare lanes 3, 5, 6, 7, 8 with lanes 11, 13, 14, 15, 16 in panel (B). Similarly, a double mutation of the transposon-specific positions does not significantly affect liposome binding either. Compare lanes 3, 5 with lanes 9, 11 in panel (C). However, the interaction with liposomes depends on the structural integrity of the ZfL2-1 esterase and not only on its charge, because heat-denaturation (d) abolishes the interaction. Compare lanes 3, 4 with lanes 11, 12 in panel (B) as well as lanes 3, 4 with 9, 10 and lanes 5, 6 with lanes 11, 12 in panel (C). The asterisk denotes a weak ES dimer in the flotation fraction, a likely gel separation artifact.

(**D**) Electrostatic potential mapped onto the molecular surface of the ZfL2-1 esterase. The modeled palmitate (Figure 3F, magenta) is included for orientation. Potentials are contoured from -5 kT/e (red) to +5 kT/e (blue). Left; top view of the putative membrane binding surface. Right; bottom view. For a side view see Figure 3G.

References

32. Vollmer, B., Schooley, A., Sachdev, R., Eisenhardt, N., Schneider, A.M., Sieverding, C., Madlung, J., Gerken, U., Macek, B. and Antonin, W. (2012) Dimerization and direct membrane interaction of Nup53 contribute to nuclear pore complex assembly. *EMBO J*, **31**, 4072-4084.

46. Jurka, J., Kapitonov, V.V., Pavlicek, A., Klonowski, P., Kohany, O. and Walichiewicz, J. (2005) Repbase Update, a database of eukaryotic repetitive elements. *Cytogenet Genome Res*, **110**, 462-467.

48. Lo, Y.C., Lin, S.C., Shaw, J.F. and Liaw, Y.C. (2005) Substrate specificities of Escherichia coli thioesterase I/protease I/lysophospholipase L1 are governed by its switch loop movement. *Biochemistry*, **44**, 1971-1979.

49. Ho, Y.S., Swenson, L., Derewenda, U., Serre, L., Wei, Y., Dauter, Z., Hattori, M., Adachi, T., Aoki, J., Arai, H. *et al.* (1997) Brain acetylhydrolase that inactivates platelet-activating factor is a G-protein-like trimer. *Nature*, **385**, 89-93.

57. Sheffield, P.J., Garrard, S., Caspi, M., Aoki, J., Arai, H., Derewenda, U., Inoue, K., Suter, B., Reiner, O. and Derewenda, Z.S. (2000) Homologs of the alpha- and beta-subunits of mammalian brain platelet-activating factor acetylhydrolase Ib in the Drosophila melanogaster genome. *Proteins*, **39**, 1-8.

70. Lupas, A. (1996) Prediction and analysis of coiled-coil structures. *Methods Enzymol*, **266**, 513-525.

71. Drin, G., Casella, J.F., Gautier, R., Boehmer, T., Schwartz, T.U. and Antonny, B. (2007) A general amphipathic alpha-helical motif for sensing membrane curvature. *Nat Struct Mol Biol*, **14**, 138-146.
